# Serial quantification of myocardial infarction tissue heterogeneity during infarct healing by cardiac MRI provides strong characterization of left ventricular remodeling (the NHLBI PROSPECT-CMR Study)

**DOI:** 10.1186/1532-429X-13-S1-O65

**Published:** 2011-02-02

**Authors:** Bobby Heydari, Shuaib M Abdullah, Otavio Coelho-Filho, François-Pierre Mongeon, Harris Alanna, Yiu Cho Chung, Rob J van der Geest, Michael Jerosch-Herold, Elliott Antman, Raymond Y Kwong

**Affiliations:** 1 Brigham and Women's Hospital, Boston, MA, USA; 2 Siemens Medical Systems, Chicago, IL, USA; 3 Leiden University Medical Center, Leiden, Netherlands

## Objective

The relationship of infarction heterogeneity with indices of ventricular remodeling quantified during the convalescent phase of acute infarct healing is unknown. We hypothesize that a dynamic increase of the proportion of infarct core relative to peri-infarct zone (PIZ) following acute myocardial infarction (MI) may be associated with adverse remodeling.

## Background

Late gadolinium enhancement (LGE) with infarct tissue heterogeneity characterized by cardiac magnetic resonance imaging (CMR) has been shown to be associated with arrhythmogenic substrates and increased mortality. Preliminary data suggests that areas of heterogeneous signal intensity (SI) within an infarct may represent an admixture of myocardial fibrosis, ischemic cellular apoptosis and viable myocytes potentially capable of functional recovery.

## Methods

We prospectively studied 63 patients with acute MI. Patients underwent CMR 2-4 weeks post MI with follow-up at 6-months. Mean age was 59 ± 12 years, and 70% were male. Successful revascularization was achieved in >90% of patients.

Total LGE was divided into infarct core and PIZ by previously described full width half-maximum method. Infarct core and PIZ were defined as regions with maximal SI ≥50^th^ and <50^th^ percentile but > peak SI of remote myocardium, respectively.

## Results

Baseline and 6-month CMR characteristics are shown in Table 1.

**Table 1:** Baseline and 6 month CMR Characteristics (n=63)

	Baseline	6 month	% change	p value
Total Infarct (g)	17±14	13±12	28±22	<0.0001
Infarct core (g)	5.5±5.6	4.0±4.0	26±32	<0.0001
PIZ (g)	11.5±9	9±8	27±23	<0.0001
LVEDV (mL)	178±50	168±47	2±16	0.16
LVESV (mL)	83±38	73±31	8±21	0.005
LVEF (%)	55±10	58±8	7±12	<0.0001

**Table 2:** 

Spearman Correlation of Infarct Characteristics			
	LVEF (% change)	LVESV (% change)	LVEDV (% change)

Infarct core (% change)	0.28 (p=0.04)	0.33 (p=0.01)	0.12 (p=NS)
PIZ (% change)	0.29 (p=0.03)	-0.38 (p=0.004)	-0.30 (p=0.03)

Dynamic change of the infarct core to PIZ ratio demonstrated strong correlation with changes in LVESV and LVEF. A dynamic increase of the infarct core to PIZ ratio, even in the setting of total infarct size regression, was significantly associated with increase of LVESV and reduction of LVEF. Percent change of infarct core strongly correlated to percent change of PIZ (r=0.66, p<0.0001).

**Figure 1:**
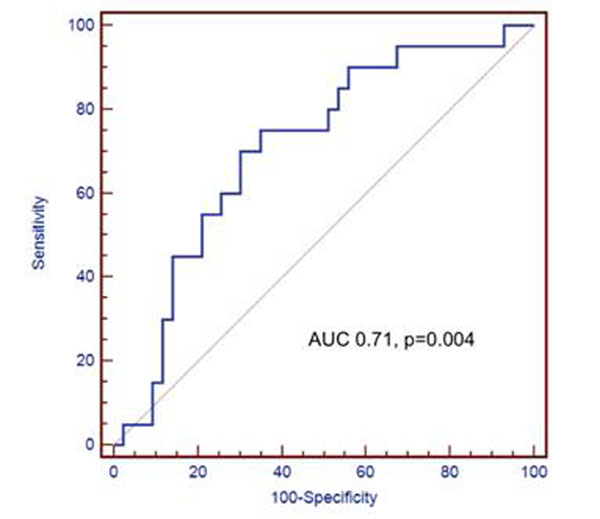
ROC of Percent Change of Infarct core/PIZ Ratio for Prediction of Adverse LV Remodeling

Figure 1 shows the receiver-operating-characteristic (ROC) curve describing the diagnostic performance of percent change of infarct core to PIZ ratio for prediction of adverse LV remodeling, with area under the curve (AUC) of 0.71, p=0.004.

## Conclusions

Our results demonstrate that while total LGE regresses, an increase of the infarct core to PIZ ratio during the first 6 months of infarct healing was associated with adverse LV remodeling. These findings provide supportive evidence that heterogeneous infarct tissue characterized by CMR in the acute infarct setting may represent an admixture of necrotic and viable myocardium. A proportional increase of infarct core relative to PIZ following MI may represent further infarct expansion, apoptosis of viable myocytes, and reduction of the extent of recoverable myocardium.

